# Application of Crumb Rubber in Cement-Matrix Composite

**DOI:** 10.3390/ma12030529

**Published:** 2019-02-10

**Authors:** Chi-Yao Chen, Maw-Tien Lee

**Affiliations:** Department of Applied Chemistry, National Chia Yi University, No.300 Syuefu Rd., Chiayi City 60004, Taiwan; ppicc1478@gmail.com

**Keywords:** partial oxidation, crumb rubber, rubcrete, waste tires, recycling, AFM

## Abstract

Many studies have used rubber as an additive to form a cement-matrix composite (rubcrete). However, rubcrete has a lower mechanical strength than standard concrete. To improve the properties of rubcrete, this study performed surface modifications on crumb rubber through a partial oxidization reaction. The optimal ratio of air to nitrogen was determined by experiments to be 1:4. Fourier transform infrared spectroscopy (FT-IR) was used to identify the functional groups on the surface of the crumb rubber. A colloidal probe of calcium silicate hydrate (C–S–H) was prepared, and the intermolecular interactions between the rubber and the C–S–H were measured using an atomic force microscope (AFM). The experimental results showed that the partially oxidized crumb rubber contained more hydrophilic S–O bonds. The intermolecular force between C–S–H and treated rubber increased by 23% compared to the force between the original rubber and C–S–H. The compressive strength of the hardened cement paste (56 days) with the treated crumb rubber increased 50% in comparison with that of the hardened cement paste with the as-received crumb rubber.

## 1. Introduction

A large number of waste tires are generated worldwide every year [1]. Tires are not biodegradable, the necessary periods for degradation are too long to be considered practically feasible. Therefore, the development of other new uses has been the focus of numerous studies. Many researchers [2,3,4,5,6] have suggested promising options to for this rubber, such as (1) creation of mixtures of rubber, asphalt, and cement; (2) generation of fuel; and (3) reuse. In Taiwan, scrap tires have been used as a fuel for cement kilns and as feedstock for making carbon black. Rubber has a high capital investment, and while using tires as a fuel is technically feasible, it is not very economically or environmentally attractive [7,8].

It is well known that crumb rubber can be used as an additive to form a cement matrix composite known as rubcrete. Rubcrete has the ability to absorb a large amount of energy under compressive and tensile loads [9,10,11,12], so it is recommended for use in circumstances where vibration damping is required, such as in shock absorbers or sound barriers in highway construction, and as an earthquake shock-wave absorber in buildings [9,10,13].

Because rubcrete is a useful material, many studies have focused on modifying crumb rubber to improve it as a concrete additive. However, because the hydrophobic crumb rubber does not distribute well in the hydrated cement and it does not bond firmly with the cement hydrate, rubcrete has a lower mechanical strength than concrete [14,15,16,17,18,19,20]. To promote the use of rubcrete, the mechanical strength of rubcrete needs to be improved [1].

Lee et al. [15] suggested that the loss of mechanical strength may be improved by a proper surface treatment of the crumb rubber. Segre and Joekes [21,22] suggested that rubber particles with rough surfaces may have improved bonding with the cement matrix. Chou et al. [23] showed that an NaOH treatment enhanced the adhesion of tire rubber particles to the cement paste. Thus, the mechanical properties of rubcrete with the NaOH-treated rubber crumbs were improved over those of the rubcrete with the as-received crumb rubber. Siddique and Naik [3,24] gave a detailed review on this topic.

To improve the compressive strength of rubcrete [25,26], most studies have tried to modify the surface properties of the rubber particles for enhanced adhesion to calcium silicate hydrate (C–S–H) colloidal probes. Chou et al. [24] showed that the adsorption of waste organic sulfur compounds on the rubber surface could enhance the hydrophilicity of the rubber and was helpful in improving the intermolecular interactions between the rubber and C–S–H. Chou et al. [27] also prepared mortars with partially oxidized crumb rubber and found that the compressive strength of the rubberized mortars increased 10% in comparison with that of the controlled mortar.

They suggested that the presence of sulfur compounds on the surface of the treated rubber may increase the strength of the rubberized mortars with the partially oxidized crumb rubber.

In this study, to prevent any effect from sand, rubberized cement pastes (instead of mortars) were prepared to understand the effects of partial oxidation on the bonds between C–S–H moieties and the treated crumb rubber. The crumb rubber was partially oxidized to enhance its hydrophilicity. Reactions were conducted in a batch reactor under various reaction conditions. The functional groups on the surfaces of the treated crumb rubber were observed via Fourier transform infrared spectroscopy (FT-IR). The contact angles of water drops on the rubber surface were measured statically. The intermolecular interactions between the rubber and the C–S–H were measured with an atomic force microscope (AFM). Cement pastes with the treated crumb rubber and the as-received crumb rubber were prepared to observe the distribution of the crumb rubber within the cement hydrate.

## 2. Materials and Methods

**Figure 1:**
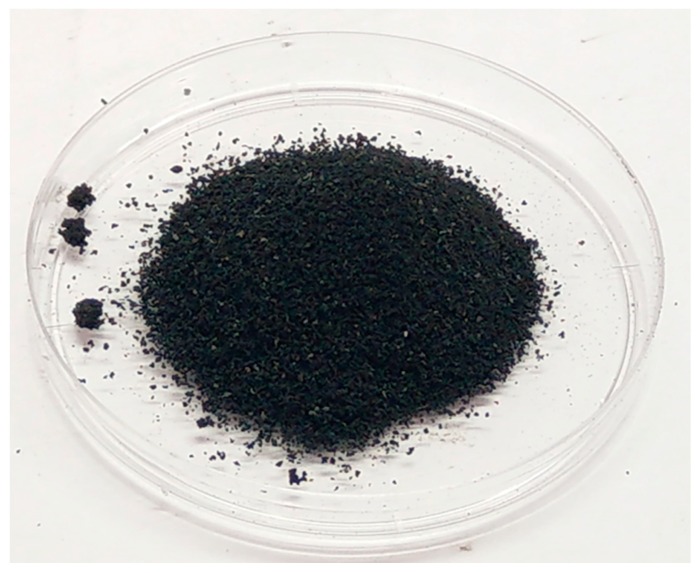
As-received crumb rubber.

Crumb rubber (styrene-butadiene rubber (SBR) + natural rubber) used in this study was supplied by the Chih Cheng Rubber Factory Co. LTD, Taiwan. The crumb rubber was screened to a particle size range of 300– 600 μm for use in the cement-matrix composite (Figure 1).

**Figure 2:**
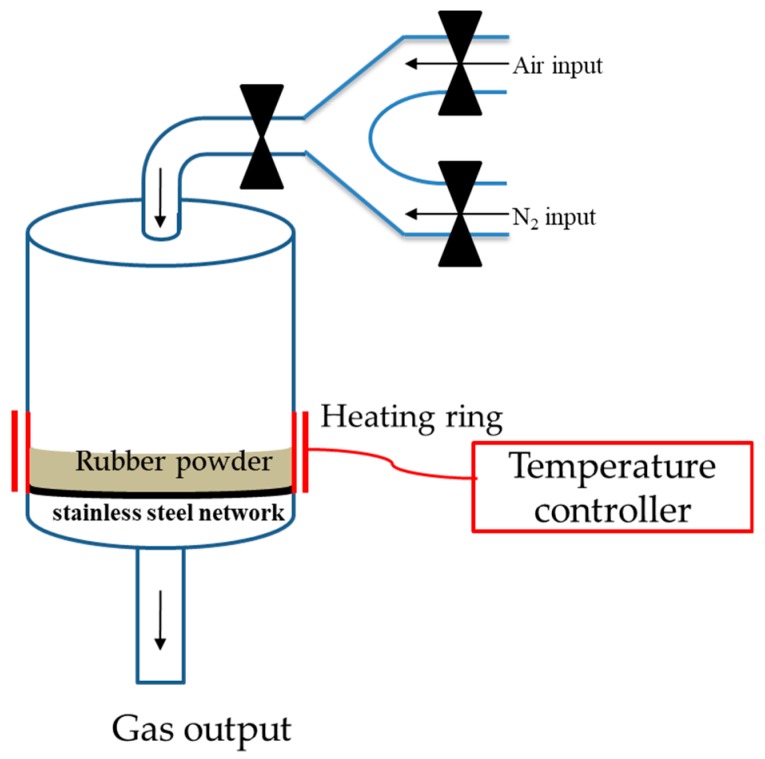
The experimental apparatus for partial oxidation of the rubber [27].

To prepare the rubber specimens for measurements of the intermolecular forces, rubber sheets (10 mm × 10 mm × 0.1 mm) were cleaned with acetone and distilled water to remove any possible contaminants on the surface. The surface roughness of the rubber specimen was observed by AFM (JPK, Axiovert 200, Berlin, Germany). To measure the intermolecular forces, a colloidal probe with C–S–H was prepared according to the method proposed by Plassard et al. [28]. Both types of rubber samples were treated by partial oxidation using the same reaction conditions. The temperature within the reactor was controlled by an electrical heating system. The flow rates of air and nitrogen were controlled and recorded to determine the air/nitrogen ratio. There were three air/nitrogen ratios: 1/0, 1/4, and 0/1. The temperatures were kept at 200 °C or 250 °C. The gas flow rate was 100 ± 10 mL/min for 30 min. The gas flows were then stopped and the temperature was raised to the desired level. Figure 2 shows the batch reactor used in this study to partially oxidize crumb rubber [27].

The change in surface functional groups of the crumb rubber was observed using FT-IR (Shimadzu FTIR-8400, Japan). The instrument “Goniometer” (Sindatek Model 100SB) was used to measure the contact angle of the rubber.

**Figure 3:**
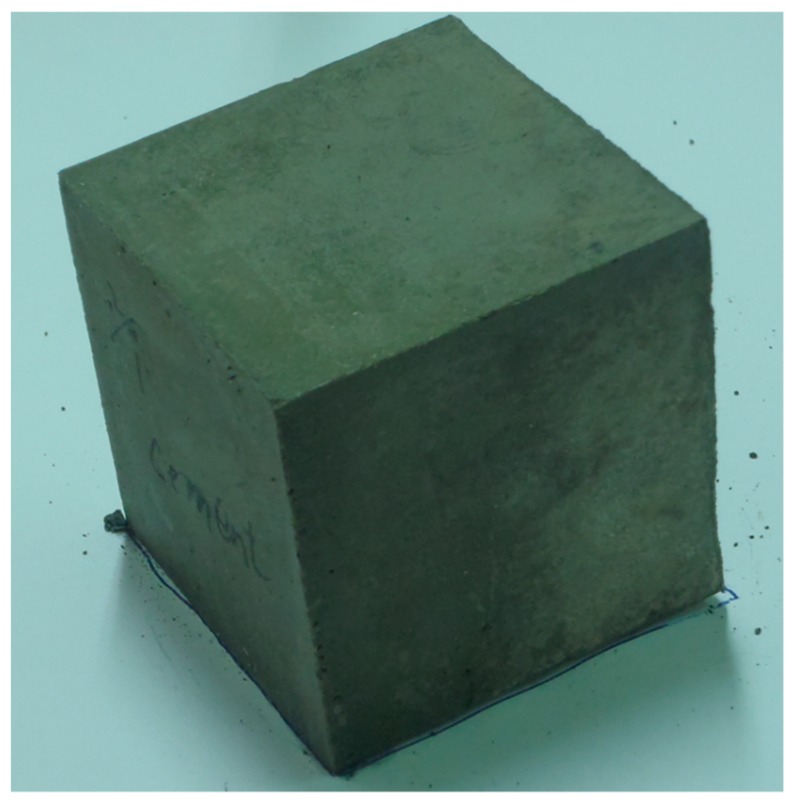
Specimen with dimensions 5 cm × 5 cm × 5 cm for compressive strength test.

Pastes with a crumb rubber level of 5% by weight were tested. The first type of Portland cement was used in this study. Specimens were prepared with a cubic molder (5 cm × 5 cm × 5 cm) to facilitate the physical testing and with water/cement 0.35 (Figure 3).

The fracture surfaces of hardened paste fragments obtained from the compressive test were observed with SEM (Hitachi SU8010, Japan) and FT-IR. The measurement of compressive strength of specimens was conducted according to ASTM standards methods, C109. Specimens aged 7 days, 14 days, 28 days, and 56 days were tested. For statistical analysis, 6 samples were used for each test. Specimens were indicated by C0, UR5, and TR5 for identification. Among them, C0 was a pure cement paste, UR5 was a paste having 5% by weight of the as-received crumb rubber, and TR5 was a paste having 5% by weight of the treated crumb rubber.

## 3. Results

### 3.1. TGA Analysis of Crumb Rubber

**Figure 4:**
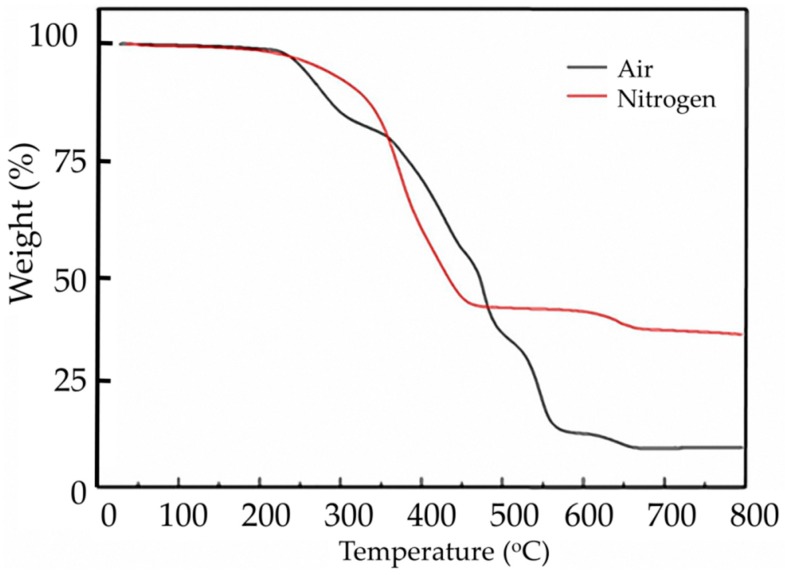
TGA curves of the crumb rubber under various ambient conditions.

The as-received crumb was analyzed by thermogravimetric analysis (TGA) under both nitrogen and air ambient to understand its characteristics to determine the reaction temperature. The results are shown in Figure 4. The first weight loss under N_2_ flow, between 200 and 300 °C [29] was attributed to the volatilization of processing oil or other low boiling-point components. The next mass loss observed in the N_2_ flow, which has a maximum rate at 350 °C, was due to the decomposition of natural rubber (NR). Since the crumb rubber used in this study contained fillers (such as carbon black and inorganic fillers), and the fillers had little mass loss at high temperature, the residue of the crumb rubber was therefore greater than that of NR. In the air, oxygen reacted with carbon black, so the residue of the crumb rubber in nitrogen was greater than that in the air (Figure 4).

Under both conditions, the two curves crossed at 350 °C. This is consistent with the results from several references [29,30,31,32]. In our experiments, we maintained the temperature of the reactor at 200 °C, and 250 °C individually to prevent from over-cracking.

### 3.2. FT-IR Spectra of Crumb Rubber

**Figure 5:**
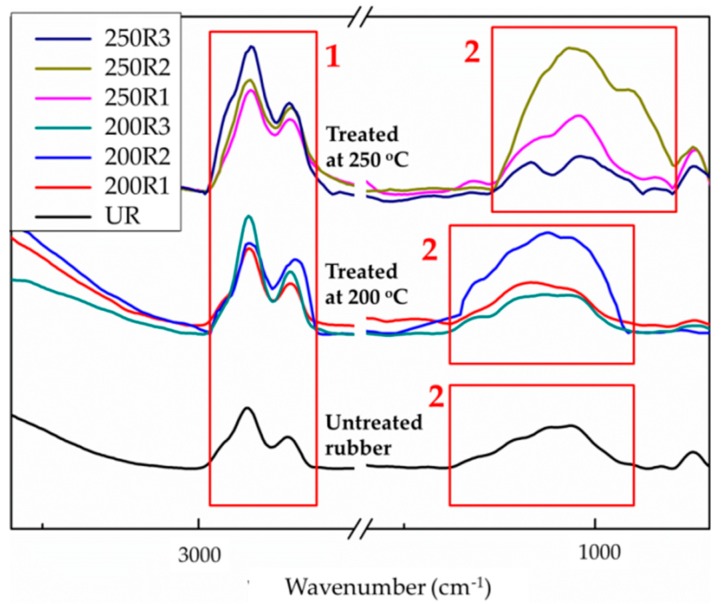
IR spectra of the crumb rubber treated at 200 °C and 250 °C. R1: air/nitrogen flow = 1/0, R2: air/nitrogen = 1/4, R3: air/nitrogen flow = 0/1, UR: as-received rubber. *1: C–H stretching, 2: SOx stretching [33].

Figure 5 shows the IR spectra of the crumb rubber treated under various reaction conditions. Where 200 and 250 are reaction temperatures (Celsius), and R represents the air/nitrogen flow ratio (R1: air/nitrogen flow = 1/0, R2: air/nitrogen = 1/4, and R3: air/nitrogen flow = 0/1). UR is the as-received rubber. The absorption in the region from 1000–1100 cm^−1^ corresponds to the stretching absorption of the two S–O bonds in the R–SO_2_–R functional groups [33]. The region from 960– 980 cm^−1^ corresponds to the S=O bond stretching absorption of the R–SO–R functionalities [33]. The region from 2800–3100 cm^−1^ corresponds to a C–H (sp^3^) stretching adsorption [33].

By treating the crumb rubber with partial oxidation, some of the R–S–R groups on the surface of the crumb rubber were oxidized to R–SO_2_–R, and R–SO–R. The production of S–O bonds on the surface of the rubber made the treated material more hydrophilic than the as-received rubber.

The area of the sp^3^ hydrocarbon bond (C–H) stretching absorption and the area of the SO_2_ and S–O (SOx) stretching absorption were integrated individually. The ratio of the SOx to C–H (sp^3^) areas in the IR spectrum was chosen as an index to evaluate the performance of the reaction, as shown in Equation (1).

(1) $$\mathrm{Area}\;\mathrm{Ratio}=\frac{SOx\;stretching\;area}{C–H\;stretchind\;area}$$

The area ratios (area of SOx/area of C–H) were calculated for the various tested air/nitrogen ratios and are listed in Table 1.

Table 1 shows that air/nitrogen = 1/4 (R2) was the optimal gas flow ratio at both tested temperatures (200 °C and 250 °C). As depicted in Figure 3, the sulfur on the surface of the rubber could be oxidized to SOx. This might be caused by oxidation of the sulfur atoms on the surface of the crumb rubber when the oxygen content present is sufficient. However, when the oxygen content is increased at high temperature, excessive oxidation of the sulfur atoms induced thermal cracking of the crumb rubber and reduced the area ratio. Chou et al. [27] suggested that the partial oxidation reaction should be conducted at or below 250 °C with an oxygen/nitrogen ratio of 0.04 or less. Based on this result, an air/nitrogen gas flow of 1/4 was chosen for the subsequent experiments.

### 3.3. Effect of Partial Oxidation on the Wettability of Rubber Surfaces and the Intermolecular Forces

**Figure 6:**
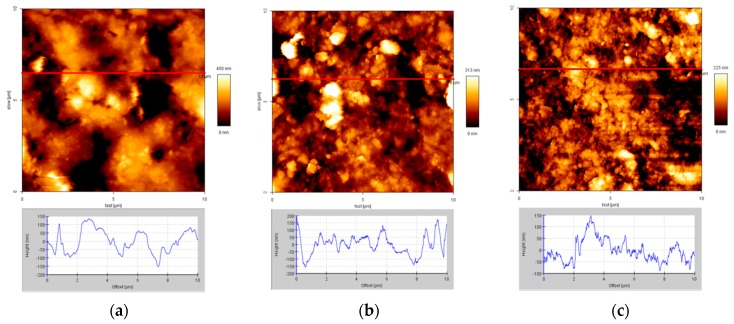
Atomic force microscopy (AFM) images (10 μm × 10 μm) and cross-section height profiles at: (**a**) The as-received rubber; (**b**) rubber treated at 200 °C with air/nitrogen = 1/4; and (**c**) rubber treated at 250 °C with air/nitrogen = 1/4.

The surface morphology of the rubber is shown in Figure 6. The treated rubber particles have a rougher, rippled surface, which could improve bonding with the cement matrix as described by Segre and Joekes [21,22].

It is well-known that rubber is a hydrophobic polymer and hydration products of cement are hydrophilic inorganic materials. The hydrophobic materials have a large contact angle and low wettability. The main interaction forces between the hydrophilic and hydrophobic materials are van der Waals forces, which are very weak. With the low interaction forces between C–S–H (the most important product of cement hydration) and rubber, the compressive strength of the cement-matrix composite containing crumb rubber will be much less than that of cement-matrix composite without crumb rubber.

### 3.4. Contact Angles

**Figure 7:**
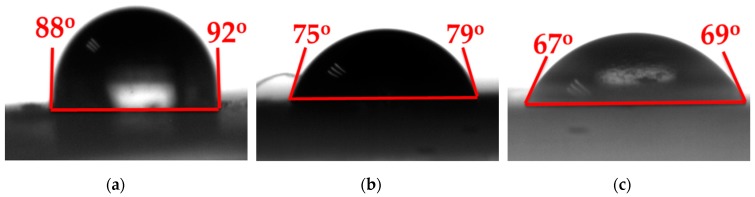
Contact angles of the (**a**) as-received rubber; (**b**) rubber treated at 200 °C with air/nitrogen = 1/4; and (**c**) rubber treated at 250 °C with air/nitrogen = 1/4.

Results of contact angle measurements are presented in Figure 7. The treated rubber has a lower contact angle than that of the as-received rubber. It was apparent that the partial oxidation treatment reduced the contact angle and, thus, enhanced the hydrophilicity of the rubber surface. It has been previously shown that enhanced hydrophilicity of the rubber surface increases the local water availability for the hydration of cement [23]. An increase in the water availability can prevent local imperfections in the hydration of the cement by the addition of heterogeneous rubber particles.

### 3.5. The C–S–H Colloidal Probe

**Figure 8:**
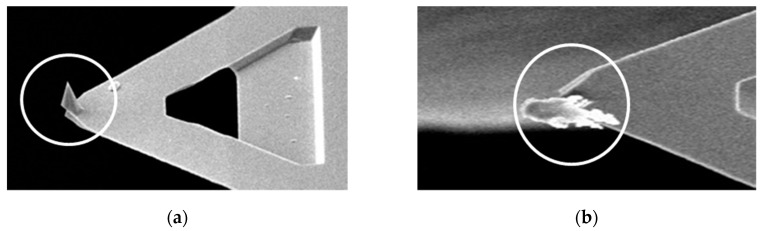
AFM probe. (**a**) Original probe; and (**b**) with C–S–H at the apex.

Figure 8 shows a colloidal C–S–H probe, and Figure 8a shows the original probe for comparison. The colloidal C–S–H probe grew at the apex of the probe, as shown in Figure 8b.

### 3.6. Intermolecular Interactions

**Figure 9:**
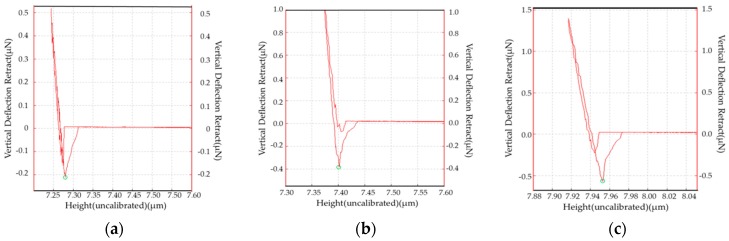
A typical intermolecular interaction force between the C–S–H probe and the rubber, measured with AFM. (**a**) As-received rubber; (**b**) rubber treated at 200 °C with air/nitrogen = 1/4; and (**c**) rubber treated at 250 °C with air/nitrogen = 1/4.

**Figure 10:**
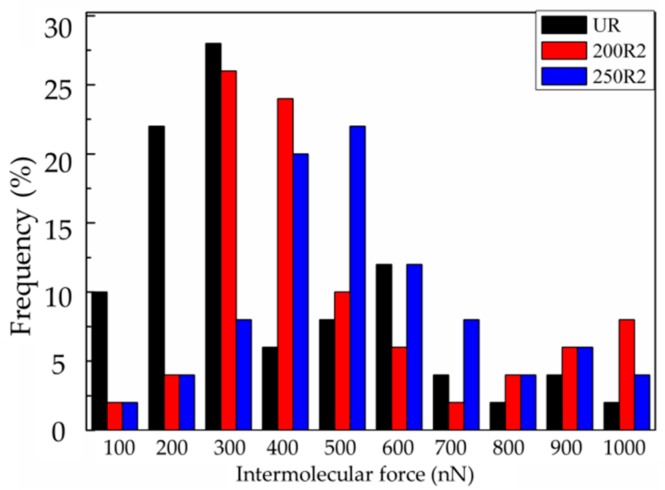
The intermolecular interaction forces between the C–S–H probe and rubber: as-received (**black**); treated at 200 °C with air/nitrogen = 1/4 (**red**); treated at 250 °C with air/nitrogen = 1/4 (**blue**).

The intermolecular interactions between the C–S–H colloidal probe and the rubber were measured via AFM, as-received rubber showed the smallest forces (Figure 9a). Furthermore, the rubber treated by partial oxidation clearly had stronger interactions with the C–S–H than the as-received rubber while the typical force-distance relationship is shown in Figure 9. The force distributions of the rubbers treated under various reaction conditions is shown in Figure 10. It was clear that the rubber treated at 250 °C displayed the largest intermolecular forces, and the as-received rubber showed the smallest forces.

### 3.7. The SEM Image of the Rubber and Cement

**Figure 11:**
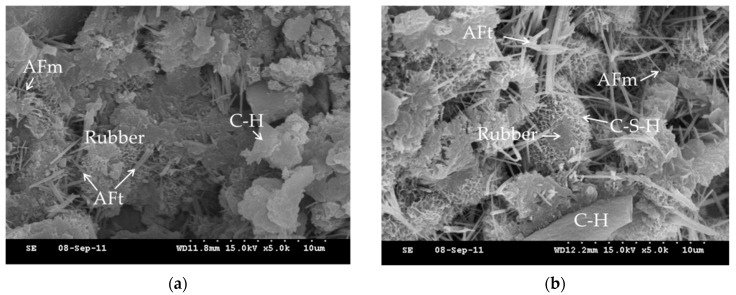
The SEM image of the additive to the cement (**a**) As-received rubber (5,000×), (**b**) treated rubber (5,000×).

Pastes with crumb rubber were observed with SEM to understand the effects of partial oxidation on the hydration of the cement. There are many kinds of cement crystal, such as calcium hydroxide (C–H), calcium monosulfoaluminate (AFm), ettringite (Aft), and calcium silicate hydrate (C–S–H) which are more representative. The SEM images are shown in Figure 11. These SEM images show that the calcium silicate hydrate (C–S–H) on the treated rubber is much more than the C–S–H on the as-received rubber. This proves that partial oxidation contributes to the formation of C–S–H on the rubber surface.

### 3.8. The Compressive Strength

**Figure 12:**
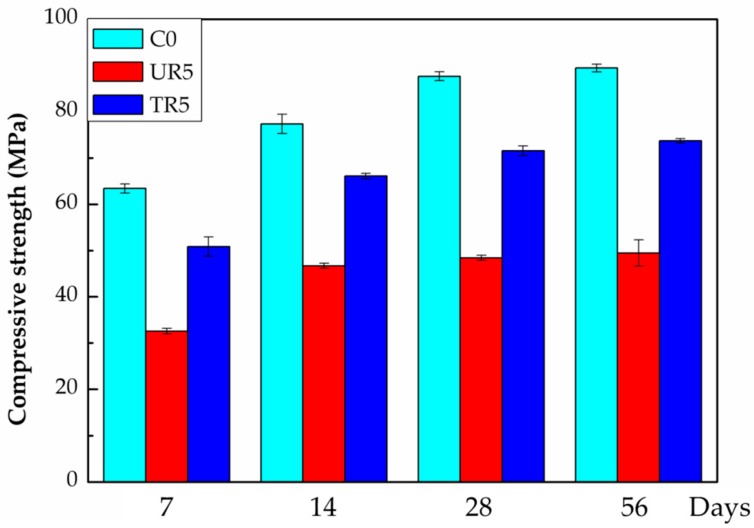
Compressive strength of hardened paste. Where C0: pure cement, UR5: with 5% as-received crumb rubber, TR5: with 5% treated crumb rubber [34,35].

The compressive strengths of the various hardened cement pastes are shown in Figure 12, where C0 represents the pure cement paste, UR5 is the cement paste with 5 wt% of the as-received crumb rubber, and TR5 is the paste with 5 wt% of the treated crumb rubber. The compressive strength of the hardened paste with the as-received crumb rubber decreased as much as 45% in comparison to that of the pure hardened paste (56 days). The compressive strength of the pure hardened cement paste is consistent with that of previous reports [34,35]. However, the compressive strength of the hardened paste with the treated crumb rubber was reduced by only 17% to 18%. The partial oxidation treatment induces the formation of hydrophilic function groups on the surface of rubber (as depicted in the IR spectra Figure 5) and therefore increases the hydrophilicity of the surface of the rubber, and the contact angle of water on the surface of the rubber decreases. It implies that the hydrophilic fresh cement paste can be spread well on the surface of the treated rubber and the hydrophilic C–S–H can also adhere firmly to the hydrophilic surface of the treated rubber. The mechanical strengths of this cement-based composite are therefore improved. These experimental results provide evidence that partial oxidation is an effective method to improve the properties of waste crumb rubber as an additive in cement-based composites.

## 4. Conclusions

The addition of rubber materials to cement mixtures decreases the physical properties of the concrete. To combat this challenge and achieve improved physical properties, partial oxidation of the rubber surface was performed in this study. The rubber treated by partial oxidation had enhanced hydrophilicity, which increased the intermolecular interactions between the rubber and a colloidal C–S–H probe, and in turn, increased the local water availability for the hydration of the cement. This increase in the water availability prevents local imperfections in the hydration of the cement on the addition of heterogeneous rubber particles. Thus, the modification of rubber surfaces via a partial oxidation reaction is a simple, economically viable process to improve the performance of rubber-concrete composites.

## Figures and Tables

**Table 1:** The SOx/C–H area ratios of the reacted rubber crumbs at 200 °C and 250 °C.

Rubber	SOx Stretching	C–H Stretching	SOx/C–H
UR	49.96	62.73	0.80
200R1	73.12	79.80	0.92
200R2	74.32	49.61	1.51
200R3	68.67	110.21	0.62
250R1	97.83	116.06	0.83
250R2	129.81	75.97	1.71
250R3	51.74	189.55	0.26

*R1: air/nitrogen flow = 1/0, R2: air/nitrogen = 1/4, R3: air/nitrogen flow = 0/1.
